# Supplement

**Published:** 1867-08

**Authors:** 


### *Supplement.*

Yielding to the solicitations of a large number of friends, we print in this No. an account of the laying of the *Corner-Stone* of Rush Medical College by the Masonic Order. As it is not strictly professional matter, we append it as a supplement, so as not to trench upon the usual pages of the JOURNAL.

